# Global phylogenomic assessment of *Leptoseris* and *Agaricia* reveals substantial undescribed diversity at mesophotic depths

**DOI:** 10.1186/s12915-023-01630-1

**Published:** 2023-06-26

**Authors:** J. C. Gijsbers, N. Englebert, K. E. Prata, M. Pichon, Z. Dinesen, R. Brunner, G. Eyal, F. L. González-Zapata, S. E. Kahng, K. R. W. Latijnhouwers, P. Muir, V. Z. Radice, J. A. Sánchez, M. J. A. Vermeij, O. Hoegh-Guldberg, S. J. Jacobs, P. Bongaerts

**Affiliations:** 1 California Academy of Sciences, San Francisco, CA 94118 USA; 2 Global Change Institute, The University of Queensland, St Lucia, QLD 4072 Australia; 3 School of Biological Sciences, The University of Queensland, St Lucia, QLD 4072 Australia; 4 Biodiversity Section, Queensland Museum, Townsville, 4810 Australia; 5 Centre for Biodiversity and Conservation Science, The University of Queensland, St Lucia, QLD 4072 Australia; 6 ARC Centre of Excellence for Coral Reef Studies, James Cook University, Townsville, QLD 4811 Australia; 7 ARC Centre of Excellence for Coral Reef Studies, The University of Queensland, St Lucia, QLD 4072 Australia; 8 The Mina & Everard Goodman Faculty of Life Sciences, Bar-Ilan University, 5290002 Ramat Gan, Israel; 9 Laboratorio de Biología Molecular Marina (BIOMMAR), Departamento de Ciencias Biológicas, Facultad de Ciencias, Universidad de Los Andes, 111711 Bogotá, Colombia; 10 Department of Oceanography, University of Hawaii at Manoa, 1000 Pope Road, Honolulu, HI 96822 USA; 11 CARMABI Foundation, Piscaderabaai Z/N, PO Box 2090, Willemstad, Curaçao; 12 Institute for Biodiversity and Ecosystem Dynamics, University of Amsterdam, Science Park 700, 1098 XH Amsterdam, The Netherlands; 13 Department of Biological Sciences, Old Dominion University, Norfolk, VA 23529 USA

**Keywords:** Mesophotic, Phylogenetics, Scleractinia, Biodiversity, Depth-differentiation

## Abstract

**Background:**

Mesophotic coral communities are increasingly gaining attention for the unique biological diversity they host, exemplified by the numerous mesophotic fish species that continue to be discovered. In contrast, many of the photosynthetic scleractinian corals observed at mesophotic depths are assumed to be depth-generalists, with very few species characterised as mesophotic-specialists. This presumed lack of a specialised community remains largely untested, as phylogenetic studies on corals have rarely included mesophotic samples and have long suffered from resolution issues associated with traditional sequence markers.

**Results:**

Here, we used reduced-representation genome sequencing to conduct a phylogenomic assessment of the two dominant mesophotic genera of plating corals in the Indo-Pacific and Western Atlantic, respectively, *Leptoseris* and *Agaricia.* While these genome-wide phylogenies broadly corroborated the morphological taxonomy, they also exposed deep divergences within the two genera and undescribed diversity across the current taxonomic species. Five of the eight focal species consisted of at least two sympatric and genetically distinct lineages, which were consistently detected across different methods.

**Conclusions:**

The repeated observation of genetically divergent lineages associated with mesophotic depths highlights that there may be many more mesophotic-specialist coral species than currently acknowledged and that an urgent assessment of this largely unstudied biological diversity is warranted.

**Supplementary Information:**

The online version contains supplementary material available at 10.1186/s12915-023-01630-1.

## Background

Because mesophotic coral ecosystems (MCEs) occur at depths beyond the limits of regular SCUBA diving (~ 30–150 m depth), they remain relatively understudied compared to shallow coral reefs, despite equalling or even exceeding the area occupied by the latter [1]. Over the past decades, interest in these deeper coral reef communities has grown due to their potential to act as a refuge against disturbances (for species with large depth distributions) [2–6] and as habitats hosting unique biological communities [7–10]. Their uniqueness is exemplified by the diversity and continuous discovery of depth-specialist fish species at mesophotic depths [7, 10], as well as the vast differences observed between shallow and mesophotic fish species assemblages [8]. Although a similar differentiation over depth has been observed for reef-building coral assemblages [8, 11], only a few scleractinian coral species dominate the assemblage at lower mesophotic depths [2, 12–17], and a small proportion of them are considered deep-specialists [17, 18].

Visual assessments (in situ or through imagery) of mesophotic coral diversity are challenging due to the intricate scale of and significant intraspecific variation in morphological traits used to identify species [18, 19]. Because collection-based assessments of coral diversity are rare for mesophotic reef corals (particularly for depths > ~60 m) [11] and because reference collections contain mostly shallow-water coral specimens [17, 20], morphological differences among shallow and mesophotic species can easily go unnoticed. Moreover, genetic identification has been hampered by the lack of species-level resolution when using traditional sequencing markers in scleractinian corals [21–24]. Consequently, these methodological challenges have greatly hindered the ability to differentiate and identify putatively new scleractinian coral species associated with mesophotic depths.

Throughout the tropics, mesophotic coral ecosystems host reef-building scleractinian coral species with predominantly plating growth forms, which maximise light capture by their symbiotic dinoflagellates to sustain photosynthesis in low light conditions at greater depths [25–27]. Most of these plating corals belong to the family Agariciidae, with the genera *Leptoseris* and *Pavona* dominating mesophotic coral communities in the Indo-Pacific, and *Agaricia* in the Western Atlantic [12, 16, 19, 28]. In these genera, species generally occur over wide depth ranges, cover large areas of substrate, and provide important habitat structure to other reef-associated organisms [15, 29, 30]. The genus *Leptoseris* is particularly abundant at lower mesophotic depths (> 60 m [26]) and has been reported down to depths of 172 m [31]. In Eastern Australia (Great Barrier Reef and Western Coral Sea), it has been observed down to 125 m depth [32], with four taxonomic species (*Leptoseris scabra* [33], *Leptoseris glabra* [20] (*Leptoseris explanata *sensu [34]), *Leptoseris mycetoseroides* [35], and *Leptoseris hawaiiensis* [33]) observed to dominate scleractinian coral communities at mesophotic depths [16, 17]. These species also have a wide geographical distribution ranging from the Red Sea [28, 36, 37] to the Hawaiian Archipelago (albeit with narrower depth distributions [19]). The Western Atlantic genus *Agaricia* comprises seven species, of which four are dominant members of mesophotic coral communities [29, 38, 39]: *Agaricia lamarcki* [40], *Agaricia fragilis* [41], *Agaricia grahamae* [42], and *Agaricia undata* [43]. These four species occur throughout most of the Caribbean basin, and *A. fragilis* even extends north to Bermuda [3] and south to the Brazilian coast [44]. The deepest *Agaricia* colony (*A. grahamae*) was reported from a depth of 119 m [45]. Nonetheless, many of these widespread *Leptoseris* and *Agaricia* species occur across large depth ranges, making them important candidates to address the question: to what extent do mesophotic coral communities harbour unique depth-specialised species?

**Fig. 1:**
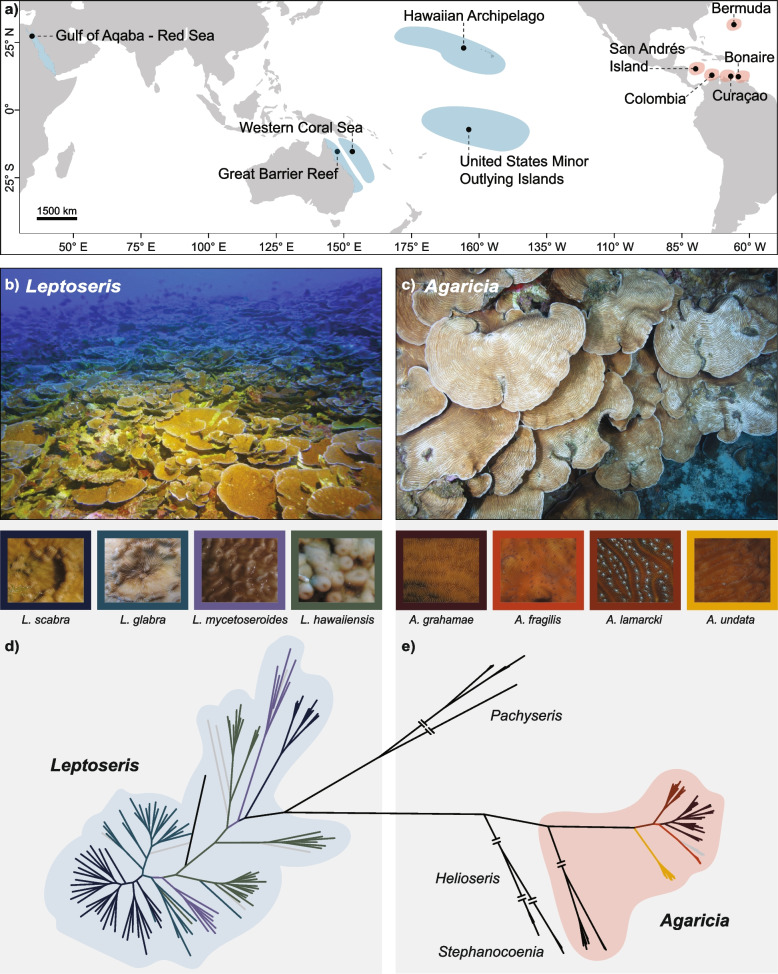
Overview of the *Leptoseris* and *Agaricia* study species. **a** Map of the sampling locations for *Leptoseris* in the Indo-Pacific (blue) and *Agaricia* in the Western Atlantic (red). **b** *Leptoseris*-dominated coral community at 87 m depth in the Hawaiian Archipelago (photo: Hawaiian Undersea Research Laboratory). **c** *Agaricia*-dominated coral community at 40 m in Curaçao, Southern Caribbean. **d** Phylogenetic tree (RAxML-ng) based on 37,528 concatenated nextRAD loci (3,361,114 sites) of the overall dataset, highlighting the position of *Leptoseris* (blue) and *Agaricia* (red) relative to included outgroups. Specimens from the focal species of this study are represented by coloured branches in the tree, with grey branches representing unidentified specimens

Molecular assessments of the genera *Leptoseris* and *Agaricia* using traditional sequence markers have exposed polyphyletic patterns, often in discordance with morphology-based taxonomy [12, 15, 19, 29, 36], and frequently were unable to discriminate between some of the well-established and morphologically distinct agariciid species [12, 21, 22, 24, 46–49]. Nonetheless, despite the pervasive and well-known issues with these markers [23, 24], the few molecular studies that have been undertaken on this ecologically important family of scleractinian corals (Agariciidae) have highlighted the potential for undescribed diversity and depth-differentiation [12, 15, 19, 36]. Reduced-representation genome sequencing methods (e.g. sequencing of restriction site-associated DNA sequencing, RAD-seq; or target capture of ultra-conserved elements, UCEs) have demonstrated their potential to overcome these issues and resulted in phylogenies that have strong support (e.g. [50–54]). Therefore, these methods are promising for studying the evolutionary relationships within the Agariciidae family. The increased resolution of such reduced representation methods was demonstrated through recent population genomics studies of *Agaricia* species, revealing significant genetic structuring within all four species dominating Atlantic mesophotic communities [3, 39, 55, 56]. Here, we build on these initial findings and present a phylogenomic assessment of the two dominant mesophotic genera found within the Agariciidae family (Fig. 1b, c) in the Indo-Pacific and Western Atlantic (Fig. 1a). Focusing on eight species from the genera *Leptoseris* and *Agaricia* (Fig. 1a, b), we evaluate whether current systematics give an accurate reflection of the species diversity and extent of specialisation (i.e. depth specificity) in mesophotic coral communities.

## Methods

### Sample collection and preparation

Coral specimens (*n* = 189) from the genera *Leptoseris* and *Agaricia* were collected as part of the “XL Catlin Seaview Survey” during visits to Eastern Australia (Western Coral Sea and Great Barrier Reef) and the Southern Caribbean (Curaçao and Bonaire; (Fig. 1a, Additional file 1: Table S1)). Samples were collected in Eastern Australia with a Seabotix vLBV300 Remotely Operated Vehicle [16, 17] and in the Southern Caribbean with the “Curasub” submersible [12] and technical SCUBA. Small fragments (~ 1 cm^2^) were stored in 99% EtOH or NaCl 20% DMSO 0.5 M EDTA solutions for genomic DNA extraction. Bleached skeletal specimens (all *Leptoseris*, and a subset of *Agaricia*) were deposited at the Queensland Museum Collection or the Invertebrate Zoology collection at the California Academy of Sciences (Additional file 1: Table S1). Additional tissue samples, acquired from collaborators, were collected from the Hawaiian Archipelago, US Minor Outlying Islands, Gulf of Aqaba (Red Sea), San Andrés Island, Cartagena, and Santa Marta (Colombia). The *Agaricia* dataset includes nextRAD sequence data (*n* = 52) from three published datasets [3, 4, 39, 55, 57, 58], and we also included *Stephanocoenia intersepta* (*n* = 3 [3, 4]), *Helioseris cucullata* (*n* = 5), and *Pachyseris speciosa* (*n* = 3 [59]) samples to serve as outgroups (Additional file 1: Table S1).

### Molecular dataset preparation

Genomic DNA was extracted as described in Bongaerts et al. [3, 60], using the additional centrifugation steps to reduce endosymbiont contamination when sufficiently high gDNA yields were obtained. Extracted gDNA was used to create nextRAD DNA libraries (SNPsaurus, LLC) using selective PCR primers to genotype genomic loci consistently [61]. gDNA was fragmented and ligated with Nextera adapters (Illumina Inc). Once ligated, fragmented DNA was PCR-amplified (26 cycles, 73 °C) with the matching primer for the adapter (“GTGTAGAGG”). Final libraries were sequenced (Illumina HiSeq 2500) to generate 100 bp single-end reads. Nextera adapters and low-quality ends (PHRED-quality score below 20) were trimmed using TrimGalore v.0.6.4 (https://github.com/FelixKrueger/TrimGalore) to discard reads < 30 bp and trim sequences up to 100 bp. IpyRAD v.0.9.62 [62] was used for locus clustering and variant calling (85% clustering threshold, minimum coverage of six, minimum four samples per final locus, with all other settings run at default recommended values) (Additional file 1: Table S2).

Symbiont contamination was identified through a BLASTN comparison of each nextRAD locus against nextRAD sequence data from isolated Symbiodiniaceae, and four published Symbiodiniaceae genomes: *Symbiodinium microadriaticum* [63], *Breviolum minutum* [64], *Cladocopium goreaui* [65], and *Durusdinium trenchii* [66] removing positive matches (maximum *E*-value = 10^−15^) from the coral nextRAD loci. Potential microbial contamination was identified by an additional BLASTN comparison against the NCBI non-redundant database. The taxonomic IDs of positive matches (maximum *E*-value = 10^−4^) of non-cnidarian taxa were removed from the dataset. Three datasets were created from the filtered assembly and used for all downstream analyses: the *Leptoseris* dataset, composed of individuals from the genus *Leptoseris* with *Helioseris cucullata* and *A. fragilis* as outgroups (*n* = 127); the *Agaricia* dataset, composed of individuals from the genus *Agaricia* with *H. cucullata* and *L. glabra* as outgroups (*n* = 70); and the “*Agalepto*” dataset, composed of individuals from both genera with *H. cucullata*, *S. intersepta*, and *P. speciosa* as outgroups (*n* = 201) (Fig. 1d, e, Additional file 1: Table S1). NextRAD loci were then trimmed to 90 bp and filtered to retain loci genotyped for ≥ 10 samples, with the resulting Variant Call Format (VCF) file filtered to retain only those SNPs genotyped for ≥ 10% of samples (Additional file 1: Table S3).

### Phylogenetic and species tree inference

Maximum likelihood (ML) phylogenetic inference was performed using RAxML-ng v.1.0.1 [67] and a concatenated matrix with complete sequences of all loci. The best fit model of nucleotide substitution GTR + I + G4 was identified by ModelTest-ng x.y.z [68] and independent searches and bootstrap replicates were performed on each alignment until convergence was reached. To assess genealogical concordance, we used the concordance factor analysis as implemented in IQ-Tree 2.1.4-beta [69, 70]. This analysis first infers single locus phylogenies (coupled with model selection) and then calculates the percentage of gene trees and alignment sites that are concordant with a reference ML topology (Gene (gCF) and site concordance factors (sCF), respectively; Additional file 1: Table S4-S5). The reference ML topology was inferred in IQ-Tree using the concatenated matrix, applying an edge-linked proportional partition model [65] and employing standard model selection [71]. Species tree inference was performed using Tetrad, a coalescent species tree approach based on SVDquartets [72] implemented in IpyRAD v.0.9.65 [62]. This analysis constructs quartets using SNPs sampled from each locus and joins these to create a ‘supertree’ statistically consistent under the multispecies coalescent model. A single SNP was randomly sampled from each locus, all possible quartets were sampled, and 100 bootstrap replicates performed generating a majority-rule consensus tree and individual trees were used to generate a density tree.

### Genetic structure using SNP-based analyses

To assess the genetic structure across our samples while avoiding the bias of a priori species assignment, we used de novo discriminant analysis of principal components (DAPC) [73], an unsupervised dimensionality reduction method where novel genetic clusters are defined using a *K*-means clustering method and subsequently visualised using principal components analysis (PCA). The DAPC analysis was conducted in R with the Adegenet package [74], choosing PCs based upon the optimal a-score and assessing the optimal numbers of clusters running *K*-means sequentially with increasing values of *K*, starting with *K* equal to the lowest AIC and BIC and then sequentially increasing *K* up to the maximum number of clades found in the ML tree for comparison.

### Species delimitation under the multispecies coalescent

We used the DELINEATE framework [75] to assess potential species boundaries of observed genetic clusters. This approach combines elements of the tree structure, branch length, and speciation completion rate to identify species. Importantly, it integrates and differentiates both population fragmentation (i.e. the initiation of potential speciation) and speciation completion events into the analysis of diversification by comparing and contrasting “known” species from “putative” species (contributing to the speciation completion rate). For each dataset (*Leptoseris* and *Agaricia*), nextRAD loci were filtered, aiming to retain ~ 200 loci (to manage computational load) while minimising the amount of missing data and maximising the number of retained individuals. Candidate genetic populations were identified using a combination of topology (observed genetic clusters), geography, and ecology-based criteria, resulting in the finest-grain population units that could be discerned. Specifically, we considered individuals from the same ecoregion and/or depth range that were also from the same genetic cluster to be a candidate population. Consequently, individuals from outlier depths, distinct ecoregions, or unidentified specimens were considered as potentially distinct lineages and identified as additional candidate populations. Using these candidate populations, a guide tree for BP&P v.4.6.2 [76] analyses was generated using StarBeast2 v.2.7.3 [77] applying a single strict clock model, and a HKY + G model of substitution. For this analysis, two replicate runs were conducted for 100 million generations each until convergence (> 250 ESS) was reached. Using this guide tree, we ran BP&P in A10 mode twice to identify distinct populations units under the multispecies (termed “multi-population” in the DELINEATE framework) coalescent model, which collapses or separates populations provided in the guide tree. We applied a posterior probability threshold of 0.90 to determine population units which were then used to generate an ultrametric phylogeny of populations using StarBeast2. This time, we conducted six independent StarBeast2 runs for 100 million generations with a sampling frequency of 5000 generations. The resulting trees were merged and summarised, generating a Maximum Clade Credibility Tree (MCCT) as the summary topology. Using this summary topology, we distinguished constrained and unconstrained lineages—distinguishing well-established species from those that remain the focus of species delimitation—by identifying morphologically and ecologically cohesive clades as constrained lineages and those lineages whose species status remains unknown or controversial as unconstrained lineages.

### Comparison with traditional sequence markers

To compare the resolution of nextRAD with traditional mitochondrial markers, *cox*1-1-rRNA intron sequence data of 101 *Leptoseris*, 9 *Agaricia*, and 1 *Pavona* individuals were amplified using AGAH/AGAL primer pairs [78]. The PCR amplifications were performed following the approach of Bongaerts et al. [29]. Agarose gels were used to assess the quality of the PCR products, then cleaned (ExoSAP-IT) and sequenced in forward and reverse directions (ABI BigDye Terminator chemistry, Australian Genome Research Facility). Additionally, previously published *cox*1-1-rRNA intron sequence data of 46 *Leptoseris* individuals [79], 61 *Agaricia* [12, 13, 80, 81], 1 *Pavona clavus* [12, 13], and 1 *L. hawaiiensis* [12, 13] individuals were retrieved from GenBank. Codoncode Aligner was used to analyse the resulting sequences. ML phylogenies were inferred using RAxML-ng v.0.9.0 on the concatenated alignment, under the K80 + G4 model, with a calculation of 20 trees and bootstrap support values based on 50,000 and 3300 replicates for species from the genera *Leptoseris* and *Agaricia,* respectively.

## Results

### Phylogenomic patterns across the genera *Leptoseris* and *Agaricia*

Using a reduced-representation sequencing approach (nextRAD), we recovered an average of 2.6 million reads (range: 580 K–12.7 M) from 201 scleractinian coral specimens; *Leptoseris* samples averaged 1.8 million reads (Additional file 2: Figure S1b), and *Agaricia* samples averaged 3.7 million reads (Additional file 2: Figure S2b). A maximum likelihood phylogenetic tree of the overall dataset, including outgroup genera (based on 37,528 nextRAD loci), confirmed that all of the ingroup specimens belonged to either the *Leptoseris* or *Agaricia* clade (Fig. 1d, e). One exception was a group of five presumed “*Leptoseris*” specimens from the Red Sea that grouped with the *Pachyseris* outgroup and were later identified as *Pachyseris inatessa* (Fig. 1e). After separating the datasets and filtering, the *Leptoseris* dataset consisted of 15,250 nextRAD loci and 100,270 SNPs, and the *Agaricia* dataset of 19,902 nextRAD loci and 221,031 SNPs. For the genealogical concordance analysis, we inferred phylogenies for all loci containing data for ≥ 10% of the samples, resulting in 10,317 and 30,650 nextRAD loci for *Leptoseris* and *Agaricia*, respectively (Additional file 2: Figure S3c-de; S4c-de). The phylogenetic analyses identified the current taxonomic species within both genera and exposed genetic substructure with additional molecular clades observed within our focal *Leptoseris* and *Agaricia* species, respectively.

For the genus *Leptoseris*, maximum likelihood phylogenetic inference using both RAxML-ng and IQTree recovered extremely similar topologies consisting of many well-supported clades, particularly at deeper nodes (RAxML-ng: 6.92–100%, bootstrap range; IQTree: 35–100%; Additional file 2: Figure S3a-b). Gene (gCF) and site concordance factors (sCF) across the IQTree topology were lower and variable, particularly near the shallow nodes of the tree (gCF: 7.0 ± 12.2%, mean ± SD; sCF: 42.1 ± 16.8%; Additional file 2: Figure S3c-d), indicating the presence of discordant signal across loci and sites (most likely attributed to the short length of nextRAD loci). The species tree analysis using Tetrad recovered similar clades as those identified with ML, although the relationship among clades differs, and their support varies (1–100%; 42.3 ± 30.4%; Additional file 2: Figure S5a). Clustering analysis (de novo DAPC) recovered similar groupings, but it identified more substructure than the latter at higher numbers of K corresponding with the same clades found in the ML and species tree (Additional file 2: Figure S6a). The exception was one group observed in a *L. glabra* clade (with mixed assignment to a spurious cluster that lacked individuals fully assigned to it). Four genetic clusters observed within the focal species were assigned by DELINEATE as putatively different species (i.e. the boundaries between them and closely related lineages were determined to be species- rather than population-level boundaries) (Additional file 2: Figure S9).

**Fig. 2:**
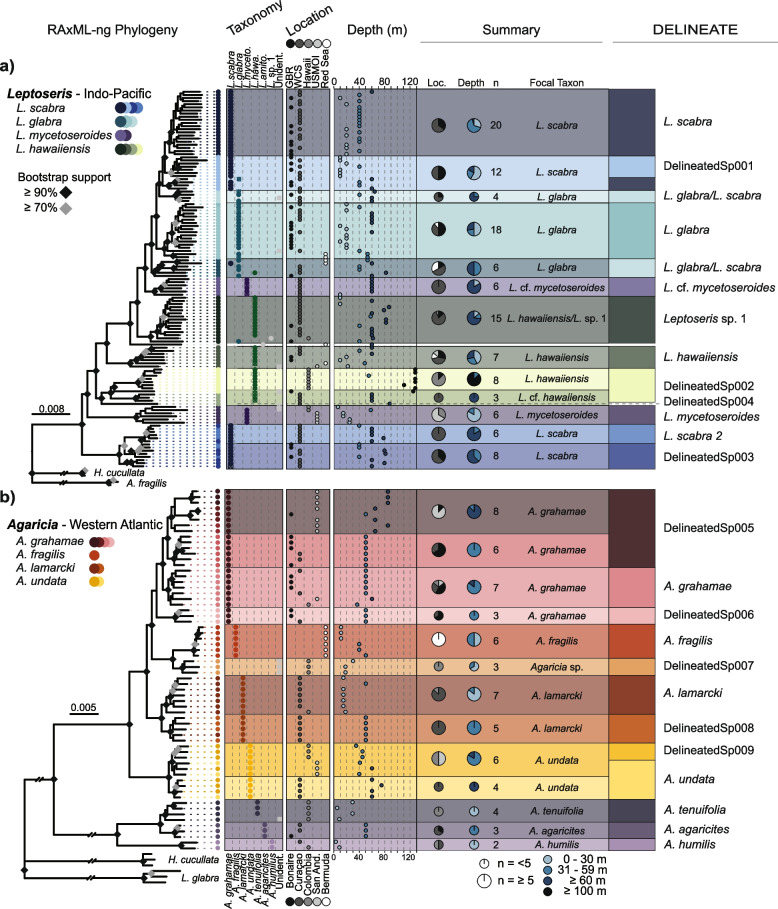
*Leptoseris* and *Agaricia*. **a** Phylogenetic tree (RAxML-ng) of the genus *Leptoseris* based on 15,250 concatenated nextRAD loci. **b** Phylogenetic tree (RAxML-ng) of the genus *Agaricia* based on 19,902 concatenated nextRAD loci. Colours across the trees represent the different identified clades (across phylogenetic and clustering methods) using blue, turquoise, purple, and green colours to represent the taxonomic species and shades of the different subclades. Columns next to the tree correspond to the taxonomic identification, sampling location, and sampling depth. The summary column on the right outlines the consensus taxonomic identification of each clade, and pie charts summarise the sampling depths and locations. The right-most column indicates the assignment based on the DELINEATE analyses (more details in Additional file 2: Figure S9; S10)

While the clades recovered across methods for *Leptoseris* were largely composed of a single taxonomic species, all taxonomic focal species were represented by multiple clades. In several cases, these different clades were separated by relatively deep nodes (e.g. for *L. scabra* and *L. mycetoseroides*; Fig. 2a), whereas others represented substructuring within major clades (based on the tree topologies, the signatures of admixture at higher values of K from DAPC analysis; Additional file 2: Figure S6a, and support for species boundaries in the DELINEATE analysis; Additional file 2: Figure S9). Samples belonging to the two divergent clades of *L. mycetoseroides* corresponded to mesophotic samples from WCS (Western Coral Sea; 5 out of 6) and shallow samples from Hawaii/USMOI (United States Minor Outlying Islands; 5 out of 6), respectively (Fig. 2a). The former clade was identified as *L.* cf. *mycetoseroides* due to morphological variations from typical *L. mycetoseroides* (Pichon and Dinesen, personal observation). Similarly, while the two clades of *L. scabra* occurred sympatrically on reefs of the WCS and GBR (Great Barrier Reef), the species delimitation analysis supports them as two putatively separate species (“DelineatedSp001”/clade A2; and “DelineatedSp003”/clade D2, Additional file 2: Figure S9). These clades were differentially characterised by depth, one clade (clade A2) was composed of a mix of shallow (10–20 m; *n* = 13) and mesophotic (40–60 m; *n* = 19) specimens and the other (clade D2) exclusively composed of mesophotic specimens (40–80 m, *n* = 14). There were three clades composed of *L. glabra* specimens; two (clade B1) had a wide geographic distribution (including Australia and Red Sea) and exclusively contain specimens from mesophotic depths (*n* = 4 and 6), and the third largest clade (clade B2; also observed in Australia and Red Sea) represented a mix of shallow (10–20 m; *n* = 8) and mesophotic specimens (40–60 m, *n* = 10). The position of the former two smaller clades was not consistent across ML and coalescent phylogenies and had inconsistent assignments in DAPC. One of the candidate populations in the bottom clade (clade B1) was therefore constrained separately as a “*L. glabra*/*L. scabra*” clade, which DELINEATE collapsed with the other clade (Additional file 2: Figure S9). *L.* (cf.) *hawaiiensis* specimens were observed across four clades (Fig. 2a). One clade (clade C1) comprised samples from primarily lower mesophotic depths on the GBR and WCS, but matched a specimen identified as *Leptoseris* sp. 1 from Hawaii [79]. A second clade (clade C2) had widespread representation containing specimens from Australia, USMOI, and the Red Sea, and originating from depths down to 60 m. In contrast, the third *L. hawaiiensis* clade (clade C3) contained almost exclusively specimens from below 120 m depth in Hawaii, with a closely related individual originating from the GBR collected at 124 m depth, and was identified as a putatively different species by the DELINEATE analysis (“DelineatedSp002”/clade C3) (Additional file 2: Figure S9). The fourth clade was identified as *L.* cf. *hawaiiensis* due to morphological variations from typical *L. hawaiiensis* (Pichon and Dinesen, personal observation), with samples from 60 m in the WCS. These were grouped together with the latter “deep” clade (clade C3) by the DELINEATE analysis (Additional file 2: Figure S9). There were also five *Leptoseris* sp. that could not be identified further: one of them (87 m depth; WCS) was related to the former *L.* (cf.) *hawaiiensis* clades. Two samples (40–60 m depth; WCS) grouped with respectively the *L. glabra* and *L. glabra/scabra* clade. The remaining two *Leptoseris* sp. originating from shallow depths (9 and 22 m, Hawaii) grouped with the shallow *L. hawaiiensis* (clade C2) and *L. mycetoseroides* clades from Hawaii; however, the former was identified as a different species in the DELINEATE analysis (“DelineatedSp004”). The analyses also included a single specimen of *L. amitoriensis* from the Red Sea, which grouped closest to the clade containing *Leptoseris* sp. 1 (Fig. 2a).

For the genus *Agaricia*, maximum likelihood phylogenetic inference using both RAxML-ng and IQTree also recovered very similar topologies consisting of several well-supported clades (RAxML-ng: 21.4–100%, bootstrap range; 80.3 ± 23.6%, mean ± SD; IQTree: 74–100%; 98.1 ± 5.3%; Additional file 2: Figure S4). Both phylogenies supported a major division of the currently acknowledged *Agaricia* genus into two major clades, representing the focal species, *A. grahamae*, *A. fragilis*, *A. lamarcki*, and *A. undata*, and the taxonomic species, *A. agaricites, A. humilis*, and *A. tenuifolia* [41] (Fig. 2b). Once again, the concordance factor values (gCF and sCF) across the IQTree topology were low and variable, particularly near the shallow nodes of the tree (gCF: 7.2 ± 12.4%, mean ± SD; sCF: 51.6 ± 22.2%; Additional file 2: Figure S4). The species tree analysis (Tetrad) recovered the same topology, consisting of the same genetic clades as those identified during ML analyses, with high statistical support, especially for the major clades (74–100%; 98.1 ± 5.3%; Additional file 2: Figure S5b). Clustering analysis (de novo DAPC) recovered similar clusters to the tree topologies and species delimitation analysis, but it also identified more substructure than the latter at higher levels of K, corresponding with the same clades found in the tree topologies (Additional file 2: Figure S6b). DELINEATE results suggest five of the genetic clusters observed across our four focal species are putatively new species (Additional file 2: Figure S10).

Although each of the observed clades in the *Agaricia* phylogenies was composed of specimens of a single taxonomic species, all four taxonomic focal species were split across two or more monophyletic subclades. *A. grahamae* exhibited further structuring beyond those subclades (also reflected in the DAPC results). *A. grahamae* specimens were represented by three major sympatric clades (Fig. 2b). Clade A1 (with substantial substructure; Additional file 2: Figure S10) corresponded to the lower-mesophotic (> 60 m) specimens from both San Andrés (*n* = 7) and specimens from Bonaire and Curaçao upper-mesophotic reefs (*n* = 7, Fig. 2b). The remaining *A. grahamae* clades (clade A2, *n* = 7; A3, *n* = 3, Additional file 2: Figure S10) consisted mostly of specimens from upper-mesophotic depths (< 60 m) from Curaçao and Bonaire, although one of those clades (clade A2) contained a sample from both Colombia and San Andrés. In the DELINEATE analysis, two of the subclades of A1 were considered separate populations of the same putative distinct species in the DELINEATE analysis (“DelineatedSp005”/clade A1), while clade A3 (“DelineatedSp006”) was classified as a putative distinct species. The *A. lamarcki* consisted of two sympatrically occurring clades with one clade exclusively representing mesophotic specimens (*n* = 6) and the other mostly shallow water specimens (*n* = 6). DELINEATE confirmed these as putatively different species (“DelineatedSp008”/clade C2). The *A*. *undata* clade was further split into two clades corresponding to two sampling regions; the Southern Caribbean (Curaçao) containing lower-mesophotic specimens (*n* = 4, clade D2) and South-western Caribbean (Colombia and San Andrés) consisting of upper-mesophotic specimens (*n* = 6), although with DAPC clustering showing admixture between clusters (Additional file 2: Figure S6b) and DELINEATE indicating species-level differentiation for the Colombian subclade (“DelineatedSp009”/clade D1). Three specimens identified as *Agaricia* sp. from Colombia formed a separate clade related to *A. fragilis* from Bermuda (*n* = 6), which was identified by DELINEATE as a putatively different species “DelineatedSp007” (clade B2). Specimens from *A. agaricites*, *A. tenuifolia*, and *A. humilis* formed a separate, highly divergent clade from the other *Agaricia* spp. Within this clade, *A. humilis* was the most divergent, with the *A. tenuifolia* from Colombia (*n* = 4) and *A. agaricites* from Curaçao and Bonaire (*n* = 3) forming more closely related groups and exhibiting high levels of admixture in the DAPC clustering analyses (Additional file 2: Figure S6b).

### Comparison with traditional sequence markers

Phylogenetic analysis of the mitochondrial sequence marker *cox*1-1-rRNA using a ML approach (RAxML-ng) for a subset of the Australian *Leptoseris* specimens (*n* = 101, Additional file 2: Figure S7) including available sequences from Luck et al. [19], identified several major molecular clades with high bootstrap support that exceeded the number of focal taxonomic species (Additional file 2: Figure S7). Even though many of these clades consisted of specimens from a single taxonomic species, substantial polyphyly was observed with clades consisting of representatives from multiple taxonomic taxa. These clades do not appear to be described by geography nor bathymetric origins, with the exception of two divergent *L. scabra* clades consisting only of mesophotic representatives (clade A1: 40–101 m, *n* = 19, clade D1/D2: 40–79 m,* n* = 7; Additional file 2: Figure S7, S9), as well as three *L. hawaiiensis* clades containing only *L. hawaiiensis* specimens mostly from mesophotic depths (clade C3: 60 m, *n* = 3; clade C1: 40–82 m, *n* = 8/9, Luck’s clade: 40–60 m, *n* = 12, including an unidentified sample from 124 m, Additional file 2: Figure S7, S9). For *Agaricia*, a comparison with available sequences (*n* = 61) from previous studies [12, 80] and additional sequences (*n* = 9) using traditional sequence markers (*cox*1-1-rRNA) distinguished between three major clades, one consisting of *A. grahamae*, *A. fragilis*, and *A. lamarcki*; one consisting of *A. undata*; and another one composed by *A. agaricites* and *A. humilis*. However, this marker did not consistently discriminate between taxonomic species within these two groups (Additional file 2: Figure S8).

## Discussion

Mesophotic coral ecosystems have gathered great scientific interest over the past decade, with numerous assessments evaluating the similarities and differences of these spatially extensive biological communities as compared to their shallow-water counterparts. From these, it has become clear that mesophotic communities become increasingly distinct with depth and can host unique and diverse species assemblages [8, 17, 82]. However, despite the growing number of accounts of depth-differentiated genetic clusters within scleractinian coral species (e.g. [3, 83, 84]), most scleractinian coral species at mesophotic depths are characterised as depth-generalists, with only a handful of reef-building scleractinian species recognised as mesophotic-specialists [18]. Through an extensive phylogenomic assessment of plating corals belonging to two dominant mesophotic genera in the Indo-Pacific and Caribbean (*Leptoseris* and *Agaricia*), we uncover undescribed diversity with (1) assumed depth-generalists representing multiple depth-associated taxa, and (2) depth-specialist species consisting of multiple sympatric taxa. Overall, the results indicate that coral communities at mesophotic depths are more speciose and likely more specialised than currently acknowledged, urging for both systematic and ecological studies to capture and better understand this diversity.

### Phylogenomic insights into *Leptoseris* diversity

The observed phylogenomic patterns based on nextRAD data confirm that *Leptoseris* represent a taxonomically diverse group and comprise several highly divergent clades. When considering assignments based on the current *Leptoseris* taxonomy [20, 34], species appear to be polyphyletic and often even separated by deep nodes (Fig. 2a), similar to previous studies using a mitochondrial intergenic spacer region [15, 19, 36]. Although the mitochondrial marker seemed initially promising for species delineation based on specimens solely from Hawaii [15, 19], it did show pervasive polyphyly that extended across both *Leptoseris* and *Agaricia* genera [19], and a lack of genetic variation across morphologically divergent taxonomic species when applied in a different geographic region [36]. Using the same marker, we obtained similar results for specimens from Australia, where some of the taxonomic diversity is captured though not consistently, with taxonomic species spread widely across the tree (Additional file 2: Figure S7). In contrast, the nextRAD data shows much more consistent phylogenetic patterns, with all recovered clades consisting almost exclusively of a single taxonomic species (Fig. 2a). The patterns corroborate the current taxonomy based on morphological differences, but with the increased genomic resolution, sample sizes, and geographic range, also unveiling additional diversity associated with specific geographic regions and bathymetric ranges. Given the congruence with both taxonomic species and spatial distributions, the observed phylogenomic patterns are expected to reflect the evolutionary relationships within this genus more closely than traditional markers and thus demonstrate the resolving power of genome-wide sequencing methods.

Within the genus *Leptoseris*, the major split in *L. mycetoseroides* seems to correspond with both geography and depth (Fig. 2a). Given that the type specimen originates from the Marshall Islands (adjacent to USMOI) [85], that there are no junior synonyms reported for this species [85], and that several specimens from East Australia were noted as morphologically distinct, it is possible that the Hawaii/USMOI clade represents the original *L. mycetoseroides* with the mesophotic specimens in the East Australian lineage representing an undescribed species. One of the lineages identified for *L. hawaiiensis* consisted of predominantly lower mesophotic specimens from the WCS and matched with a *Leptoseris* “sp. 1” specimen from Hawaii, where it was identified as a putatively new species based on its distinct micromorphology [19]. In the *cox*1-1-rRNA phylogeny, one of our specimens from this lineage also grouped with the *Leptoseris* “sp. 1” clade from Luck et al. [19], although the other specimens from the same nextRAD clade were widely spread across the tree (Additional file 2: Figure S7). This indicates that this predominantly mesophotic clade is present in Australian waters and may be geographically widespread, although further investigations are warranted. The other two *L. hawaiiensis* clades each showed different depth distributions, with one from predominantly > 100 m depth in Hawaii, and another representing morphologically atypical specimens from 60 m depth in Australia, although the DELINEATE analysis identified both as part of the same putative distinct species. Related to these clades but branching separately was a single specimen collected from the GBR at 124 m, representing the deepest published report of a zooxanthellate coral collected from the GBR [32], and indicating that *Leptoseris* lineages occurring at the lower boundaries of mesophotic depths may represent a distinct species. We observed a similar partitioning into depth-associated clades for *L. scabra*, with a further subdivision into additional geographically sympatric clades. As reported by Luck et al*.* [19], the deeper clade of *L. scabra* is more divergent compared to the other *Leptoseris* taxa (although not resulting in generic polyphyly), and with *cox*1-1-rRNA sequences matching those from Hawaii. Detailed morphological characterisation of the specimens (beyond identification according to currently acknowledged taxonomic species) was beyond the scope of the current study, but is the focus of ongoing work that aims to determine which of the exposed taxa are cryptic versus morphologically differentiated.

### Phylogenomic insights into *Agaricia* diversity

For the genus *Agaricia*, we observed a split into two major clades corresponding to species with deeper (*A. grahamae*, *A. fragilis*, *A. lamarcki*, *A. undata*) and shallower (*A. agaricites*, *A. humilis*, *A. tenuifolia*) distributions (Fig. 2b). This division was also observed using the *cox*1-1-rRNA region (Additional file 2: Figure S8) as well as other mitochondrial markers [12, 29, 76, 86–88], although these markers have lacked the resolution to consistently discern the established taxonomic species within the clades. The division into two major clades is further corroborated by a split in gross morphology (unifacial versus bifacial colonies), microskeletal characteristics (including wall thickness and septocostae orientation; [89]), and genetic relatedness among symbiont associations within each major clade [29]. Based on a morphological analysis of modern and fossil representatives, Stemann [89] proposed these should represent two separate genera: *Agaricia* and *Undaria* [90]. Recent differences in spatial genetic structure observed across taxa further suggest major differences in reproductive strategies between these major clades [55]. Given the major phylogenetic divergence observed in this study (Fig. 2b), and further corroborated by the major locus dropout between clades (Additional file 2: Figure S2), we argue that the shallower and deeper *Agaricia* indeed represent two genetically, morphologically, and biologically divergent groups that arguably might warrant generic reassignment.

The phylogenetic analyses supported the current taxonomic species within *Agaricia* but revealed genetic substructure with additional lineages observed within our four focal *Agaricia* species (Fig. 2b). This structure was consistently recovered across phylogenetic and clustering methods and often corresponded to different depths and/or geographic regions, indicating the role of both environmental and geographical (i.e. allopatric) contributors to the underlying diversification processes. Our analyses included four focal species previously assessed in population genomic studies with varying levels of intraspecific genetic structure [3, 39, 55]. Through a combined phylogenomic analysis and species delimitation approach, we were able to assess this genetic structuring in the context of interspecific variation. For example, despite the genome-wide differentiation observed for shallow and deep *A. fragilis* populations in Bermuda [3], representative specimens of those populations formed a single lineage here (i.e. under a phylogenomic framework), indicating that these reflect the earlier stages of the divergence continuum. In contrast, the *A. lamarcki* samples clearly separated into two distinct lineages associated with predominantly shallower (15 m) and upper mesophotic (50 m) depths (Fig. 2b) despite their geographically sympatric distribution and ongoing low levels of gene flow [55], and clearly represent distinct evolutionary units (Additional file 2: Figure S10). Similarly, we observed three distinct lineages of *A. grahamae* occurring sympatrically in Curaçao and Bonaire with varying levels of divergence (Fig. 2b), of which all of them were delimited as putative species (Additional file 2: Figure S9). While these lineages were not partitioned by depth, it indicates undescribed specialist mesophotic taxa that are currently not accounted for. The two phylogenetic clades observed for *Agaricia undata* corresponded with geography (Curaçao vs Colombia and San Andrés); however, DELINEATE separates these groups differently (Curaçao and San Andrés vs Colombia). Several Colombian specimens that could not be confidently identified down to species-level through morphological assessment (*Agaricia* sp. clade) grouped as a distinct lineage related to *A. fragilis* from Bermuda, indicating the presence of a related species in the Southern Caribbean. In the aforementioned “*Undaria*” clade, *A. humilis* formed a separate clade from *A. agaricites* and *A. tenuifolia* specimens, corroborating their ecological and reproductive differences [38]. The separation between *A. agaricites* and *A. tenuifolia* was confounded by distinct geographic origins for the specimens; however, the contrasting support between clustering and species delimitation analyses indicates that these morphologically similar species warrant further taxonomic investigation.

### Evolutionary patterns and remaining challenges

As a result of this study, we can now begin to directly compare and contrast the present diversity of these genera and to hypothesise the existence of associated generative processes. An evident pattern we observe across both genera is the depth-associated divergence within several focal species (e.g. *L. scabra*, *L. hawaiiensis*, and *A. lamarcki*). Depth is an important contributor to the divergence of marine species [78, 91, 92], with several well-studied examples in scleractinian corals (e.g. [93–97]). Our results corroborate the expectation that depth has led to divergent species associated with mesophotic depths [92] and that the underestimated diversity of mesophotic-specialist species is likely to be a consequence of logistical and taxonomic challenges [18]. However, depth does not immediately explain the sympatric taxa we observed within mesophotic-specialist species (e.g. *A. grahamae* (A1) and the “deep” *L. scabra* clade (D1/2), lacking clear depth differences). Their ecological differentiation may be related to finer-scale environmental conditions and could be assessed further within a spatially explicit framework to allow microhabitat characterisation [98].

Another clear pattern we observe here is the higher levels of taxonomic/phylogenetic discordance in (often sympatrically occurring) genetic groups in *Leptoseris*, relative to *Agaricia.* This difference might be the result of *Leptoseris* having a larger geographic distribution spanning a variety of ecoregions (Indo-Pacific and Caribbean; [99, 100]) in contrast to the relatively restricted geographic distribution of *Agaricia* in the Western Atlantic and Caribbean [99]. In addition, the fossil record of *Leptoseris* dates back to the Oligocene (~ 23 Ma years, [101]), while *Agaricia* is a younger genus, dating from the Neogene ~ 12 Ma years [102], and likely diversified over the last ~ 3 Ma years following the closure of the Central American Seaway [99, 102]. These differences suggest increased opportunities for ecological diversification in *Leptoseris* (compared to *Agaricia*), while signatures of admixture (Additional file 2: Figure S6a) suggest a possible history of hybridisation and reticulation (i.e. the process of genetic lineages both merging and/or diverging through time; [103]) among its lineages. It is important to note, however, that the number of loci and sites supporting some of these polyphyletic patterns are low (Additional file 2: Figure S3c); thus, additional sequence data (e.g. longer/more informative loci, whole-genome data) might reveal additional patterns within this genus (see below).

The reduced representation sequencing data across multiple analytical approaches consistently recovered groupings of individuals (i.e. clades and/or clusters) consistent with biological, ecological, or morphological evidence. Compared to traditional sequence markers, our genome-wide sequencing approach resulted in a higher resolving power, confirming the status of current morphologically and ecologically divergent taxonomic species and allowing us to gain a better insight into the evolutionary history of members of both genera. Despite the consistent topology recovered across approaches, the support for these underlying phylogenies did vary across analytical methods. For example, despite the strong bootstrap support recovered across all ML phylogenies, the gene- and site-concordance analysis revealed variable values in both datasets, indicating, on average, a low fraction of gene trees and a moderate fraction of alignment sites supporting each topology (Additional file 2: Figure S3; S4). This discrepancy between methods is often observed across different RAD-seq approaches, in most cases, due to the short length of each locus and its associated phylogenetic information [104–107]. Targeted sequence-capture approaches focused on ultra-conserved elements, or exons, can potentially represent an alternative to recover greater concordance across loci. Because conserved regions are less affected by demographic events, and next to these regions, more variable sites can be considered side by side, these genomic regions provide a more holistic view of individual evolutionary histories [108, 109]. Compared to RAD-seq, however, the generally greater cost of such sequence-capture approaches often translates to smaller sample sizes and less replication. Regardless, the implemented phylogenetic and species delimitation methods are only able to consider genetic divergence as a determinant of species status (e.g. phylogenetic species concept) and whether these groups exhibit either intrinsic or extrinsic reproductive barriers remains untested. To this end, we also considered depth, geography, and sympatry in the assessment of potential of species boundaries. We recognise the limitations and risk of circular reasoning in the used species delimitation method, especially with suboptimal sampling (per depth and geographic region) and the difficulty and associated bias of assigning the constrained lineages, and we, therefore, recommend the identified delineated species to be interpreted with caution. Ultimately, whole-genome resequencing of representatives across a wide range of depths and geographies will enable the identification of the loci involved in the diversification of *Leptoseris* and *Agaricia*, help assess divergence histories through considering introgression events, and uncover the nature of reproductive barriers if paired with environmental, phenotypic, and reproductive data.

## Conclusions

Our results shed light on the diversity of two key coral genera of mesophotic ecosystems, *Leptoseris* and *Agaricia*. Using genome-wide sequencing data in a phylogenomic framework, we observed that genomic data corroborate current morpho-taxonomic criteria, but also exposed considerable undescribed diversity associated with mesophotic depths. Distinguishing where these different taxa sit along the speciation continuum remains difficult, due to the challenges of species delimitation methods. Nonetheless, current taxonomic species were observed to comprise multiple highly divergent (e.g. *L. scabra*, *L. mycetoseroides*) or sympatrically occurring but geographically widespread lineages (e.g. *L. glabra*, *A. grahamae*), indicating that a reasonable extent of reproductive isolation has evolved. Further integrative taxonomic studies are currently being developed to formally describe the uncovered species diversity, and verify whether these taxa are morphologically cryptic, differentiated, and/or potentially align with junior synonyms. Overall, our study highlights how our perception of mesophotic coral ecosystems is affected by our shallow knowledge bias, and that studying the ecology and evolution of this newly exposed mesophotic biodiversity should be a priority in order to advance our understanding of these ecosystems.

## Supplementary Information

**Additional file 1: Table S1.** Specimens overview. **Table S2.** IpyRAD params file. **Table S3.** Data filtering. **Table S4.** Concordance factor statistics for the *Leptoseris* dataset. **Table S5.** Concordance factor statistics for the *Agaricia* dataset.

**Additional file 2:** **Figure S1.** Number of shared sites among *Leptoseris* specimens. **Figure S2.** Number of shared sites among *Agaricia* specimens. **Figure S3.** Genealogical concordance for the *Leptoseris* dataset. **Figure S4.** Genealogical concordance for the *Agaricia* dataset. **Figure S5.** Evolutionary relationships of mesophotic *Leptoseris* and *Agaricia* species. **Figure S6.** De novo clustering and ordination methods to assess genetic structure within the genus *Leptoseris* and *Agaricia*. **Figure S7.** Comparison of maximum-likelihood based phylogenies of Australian and Hawaiian *Leptoseris* specimens. **Figure S8.** Comparison of maximum-likelihood based phylogenies of *Agaricia* specimens. **Figure S9.** Species delimitation across the *Leptoseris* genus. **Figure S10.** Species delimitation across the *Agaricia* genus.

## Data Availability

Raw sequence data for reduced-representation (nextRAD) sequencing are available at the NCBI Sequence Read Archive under BioProject PRJNA970738 [110].

## Abbreviations

MCE: Mesophotic coral ecosystem
RAD-seq: Restriction site-associated sequencing
UCE: Ultra-conserved elements
VCF: Variant call format
ML: Maximum likelihood
gCF: Gene concordance factors
sCF: Site concordance factors
DAPC: Discriminant analysis of principal components
PCA: Principal components analysis
WCS: Western Coral Sea
USMOI: United States Minor Outlying Islands
GBR: Great Barrier Reef
