# Supplementary material for: Global phylogenomic assessment of Leptoseris and Agaricia reveals substantial undescribed diversity at mesophotic depths
Source: BMC Biol. 2023 Jun 26;21:147. doi: 10.1186/s12915-023-01630-1 (PMC10294349; doi:10.1186/s12915-023-01630-1)
Supplement: Supplementary file 2 — Additional file 2: Figure S1. Number of shared sites among Leptoseris specimens. Figure S2. Number of shared sites among Agaricia specimens. Figure S3. Genealogical concordance for the Leptoseris dataset. Figure S4. Genealogical concordance for the Agaricia dataset. Figure S5. Evolutionary relationships of mesophotic Leptoseris and Agaricia species. Figure S6. De novo clustering and ordination methods to assess genetic structure within the genus Leptoseris and Agaricia. Figure S7. Comparison of maximum-likelihood based phylogenies of Australian and Hawaiian Leptoseris specimens. Figure S8. Comparison of maximum-likelihood based phylogenies of Agaricia specimens. Figure S9. Species delimitation across the Leptoseris genus. Figure S10. Species delimitation across the Agaricia genus. [file 12915_2023_1630_MOESM2_ESM.pdf]

SUPPLEMENTARY FIGURES

Global phylogenomic assessment of *Leptoseris* and *Agaricia* reveals substantial undescribed diversity at mesophotic depths

Gijsbers JC[1]*, Englebert N[2], Prata KE[1,3], Pichon M[4], Dinesen Z[5], Brunner R[2,6], Eyal G[3,7,8], González-Zapata FL[9], Kahng SE[10], Latijnhouwers KRW[11,12], Muir P[2], Radice VZ[2,13], Sánchez JA[9], Vermeij MJA[11,12], Hoegh-Guldberg O[2,3,7], Jacobs SJ[1], Bongaerts P[1,2,11]*

[1] California Academy of Sciences, San Francisco, CA 94118, USA

[2] Global Change Institute, The University of Queensland, St Lucia, Queensland 4072, Australia

[3] School of Biological Sciences, The University of Queensland, St Lucia, Queensland 4072, Australia

[4] Biodiversity Section, Queensland Museum, Townsville 4810, Australia

[5] Centre for Biodiversity and Conservation Science, The University of Queensland, St Lucia, Queensland 4072, Australia

[6] ARC Centre of Excellence for Coral Reef Studies, James Cook University Townsville, Queensland 4811, Australia

[7] ARC Centre of Excellence for Coral Reef Studies, The University of Queensland, St Lucia, Queensland 4072, Australia

[8] The Mina & Everard Goodman Faculty of Life Sciences, Bar-Ilan University, Ramat Gan, 5290002, Israel

[9] Laboratorio de Biología Molecular Marina (BIOMMAR), Departamento de Ciencias Biológicas, Facultad de Ciencias, Universidad de Los Andes, Bogotá 111711, Colombia

[10] Department of Oceanography, University of Hawaii at Manoa, 1000 Pope Road, Honolulu, HI 96822, USA

[11] CARMABI Foundation, PO Box 2090, Piscaderabaai z/n, Willemstad, Curaçao

[12] Institute for Biodiversity and Ecosystem Dynamics, University of Amsterdam, Science Park 700, 1098 XH Amsterdam, The Netherlands

[13] Department of Biological Sciences, Old Dominion University, Norfolk, VA 23529, USA

* Corresponding authors: jgijsbers@calacademy.org, pim@calacademy.org

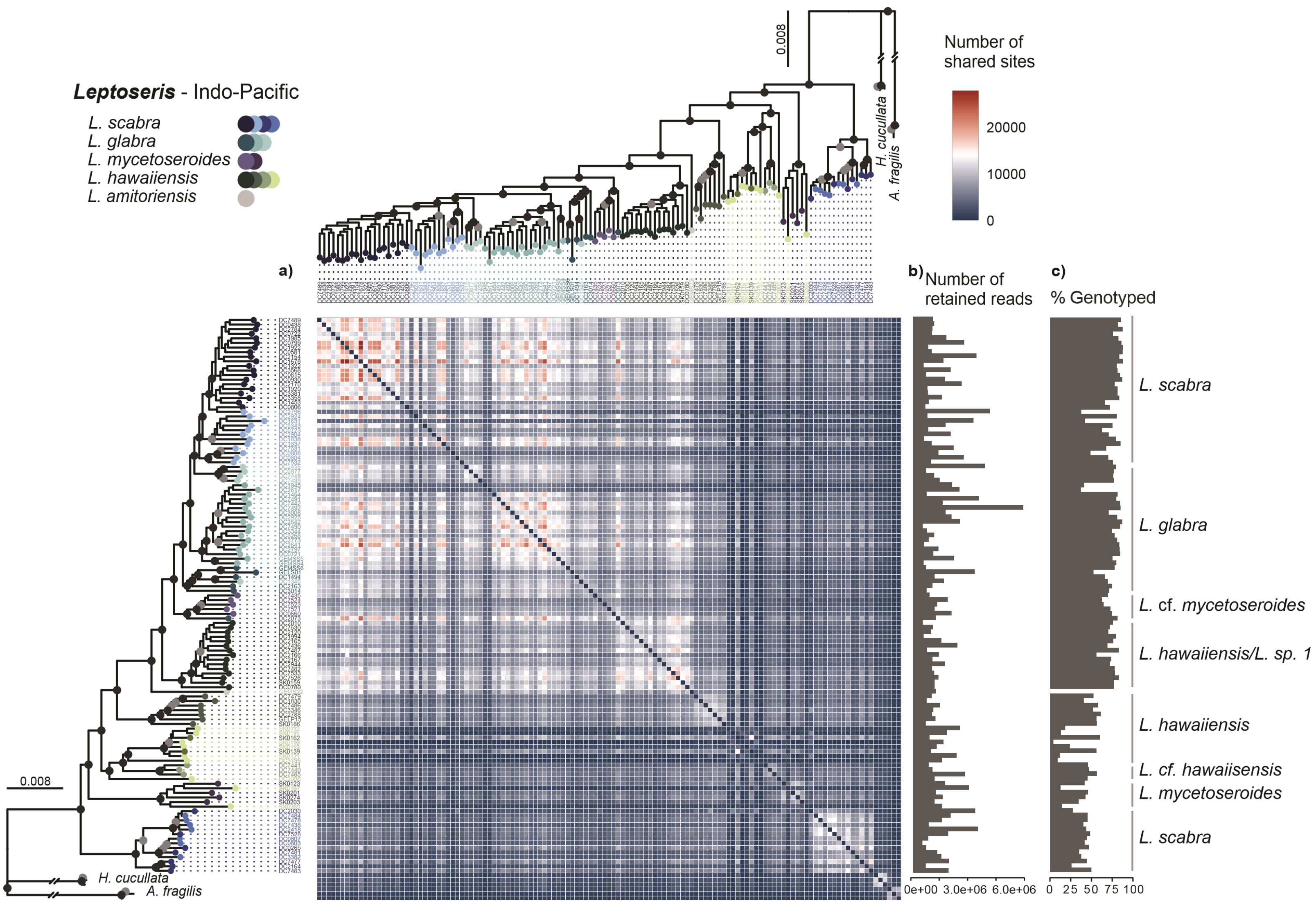

Supplementary figure S1. Number of shared sites among *Leptoseris* specimens. (a) Heat map representing the number of shared sites between individuals. Red and Blue correspond respectively, to the highest and lowest amount of shared sites between individuals. (b) Barplot representing the number of retained reads across individuals. (c) Barplot representing the percentage of genotyping across individuals.

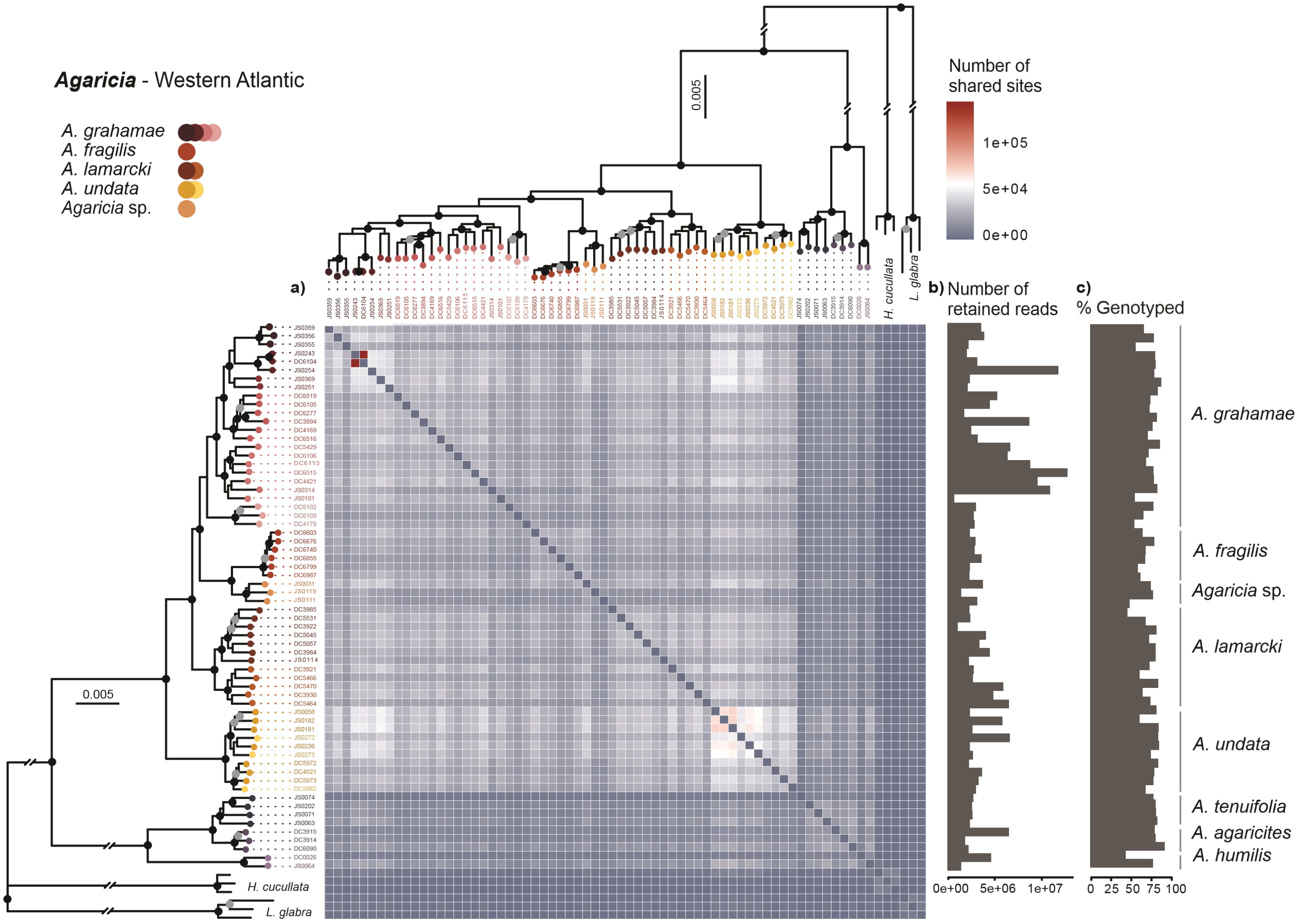

Supplementary figure S2. Number of shared sites among *Agaricia* specimens. (a) Heat map representing the number of shared sites between individuals. Red and Blue correspond respectively, to the highest and lowest amount of shared sites between individuals. (b) Barplot representing the number of retained reads across individuals. (c) Barplot representing the percentage of genotyping across individuals.

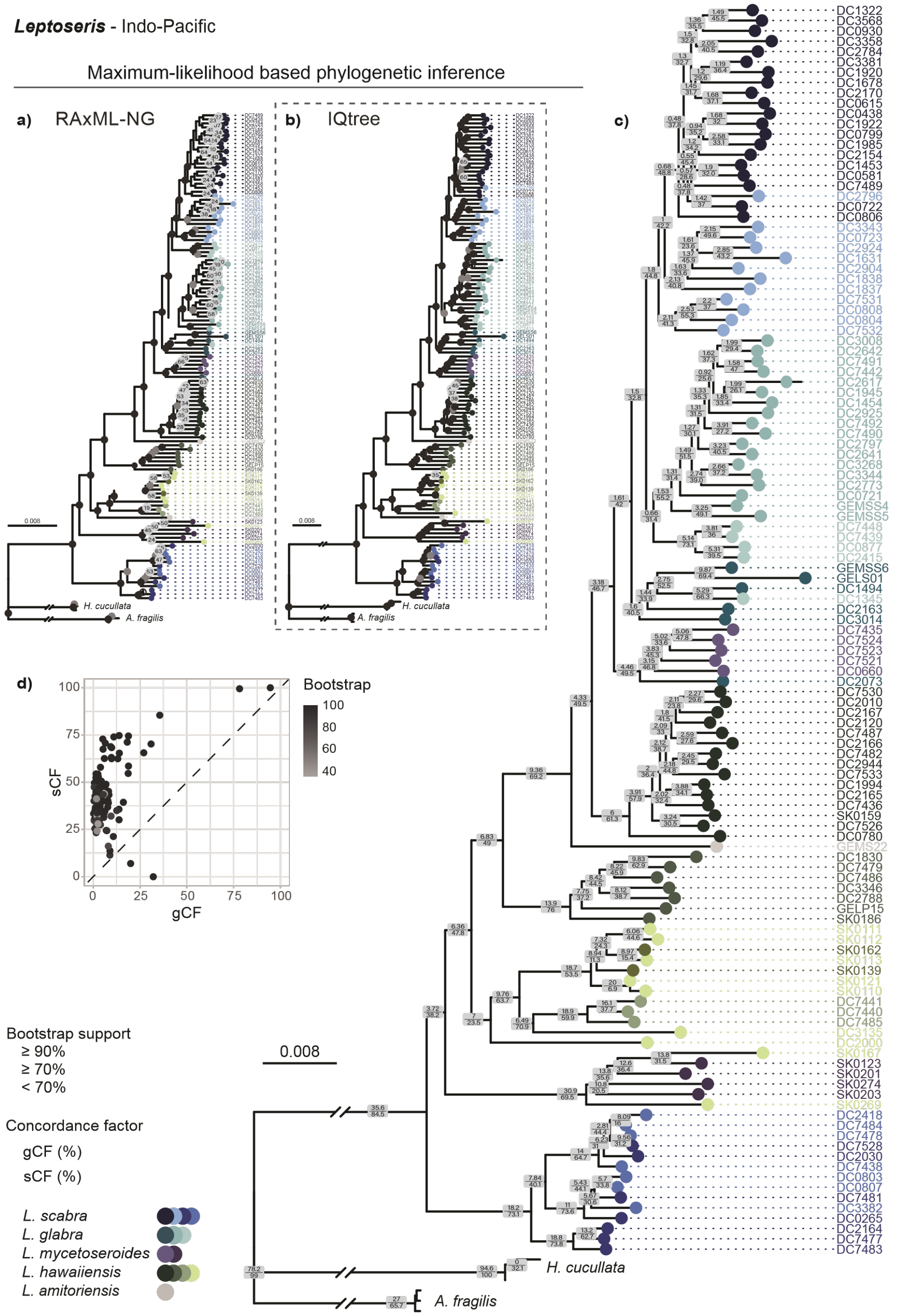

Supplementary figure S3. Genealogical concordance for the *Leptoseris* dataset. (a) RAxML-NG tree based on 15,250 concatenated nextRAD loci. (b) IQtree species tree based on 10,317 single full loci. (c) IQtree tree from inset with concordance factor values. Numbers in grey squares on each branch represent the gene concordance factor (gCF) above and the site concordance factor (sCF) on the bottom. (d) Scatter plot of sCF against gCF values in relation to the bootstrap values of 1000 ultrafast bootstrap replicates.

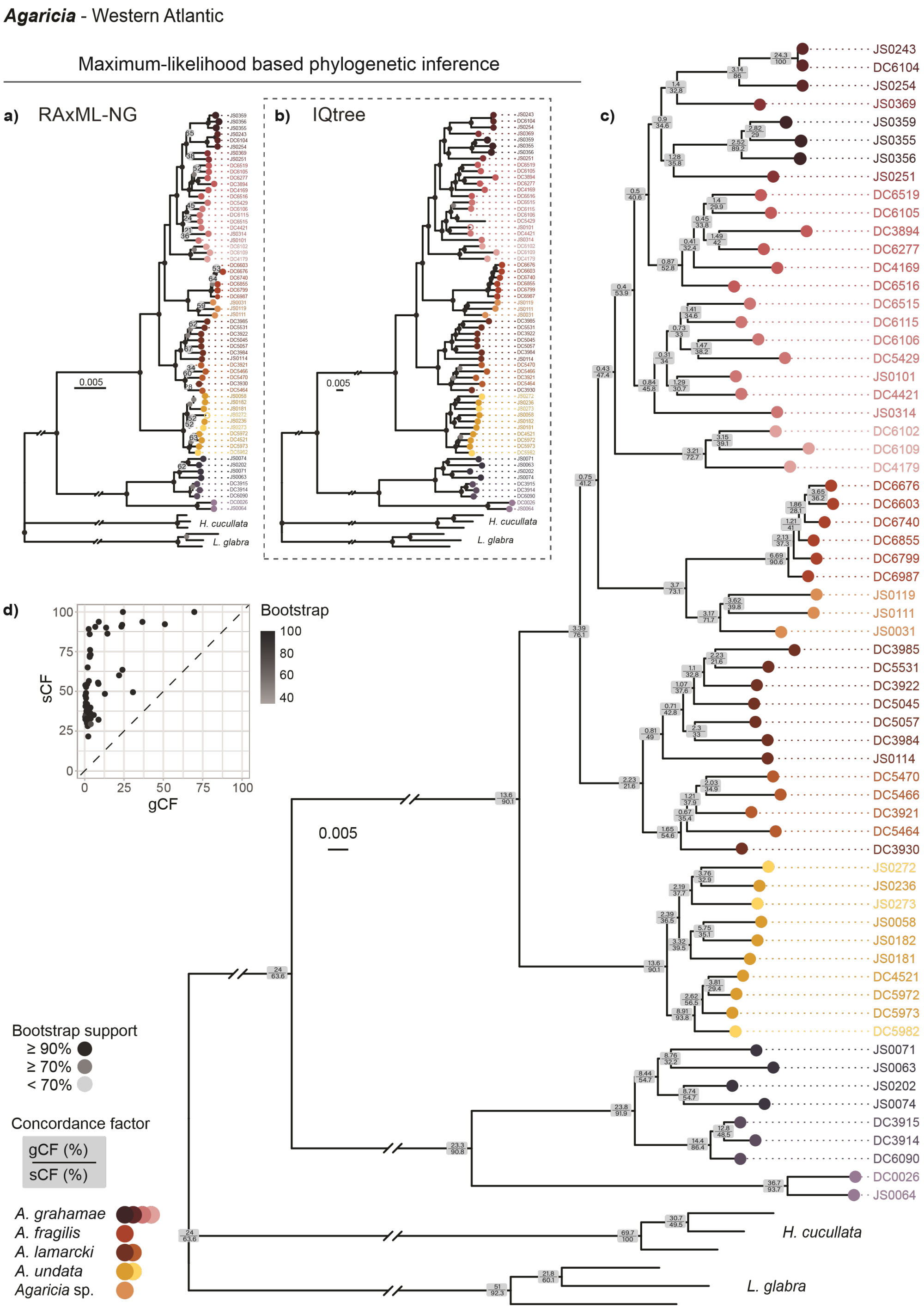

Supplementary figure S4. Genealogical concordance for the *Agaricia* dataset. (a) RAxML-NG tree based on 19,902 concatenated nextRAD loci. (b) IQtree species tree based on 30,650 single full loci. (c) IQtree tree from inset with concordance factor values. Numbers in grey squares on each branch represent the gene concordance factor (gCF) above, and the site concordance factor (sCF) on the bottom. (d) Scatter plot of sCF against gCF values in relation to the bootstrap values of 1000 ultrafast bootstrap replicates.

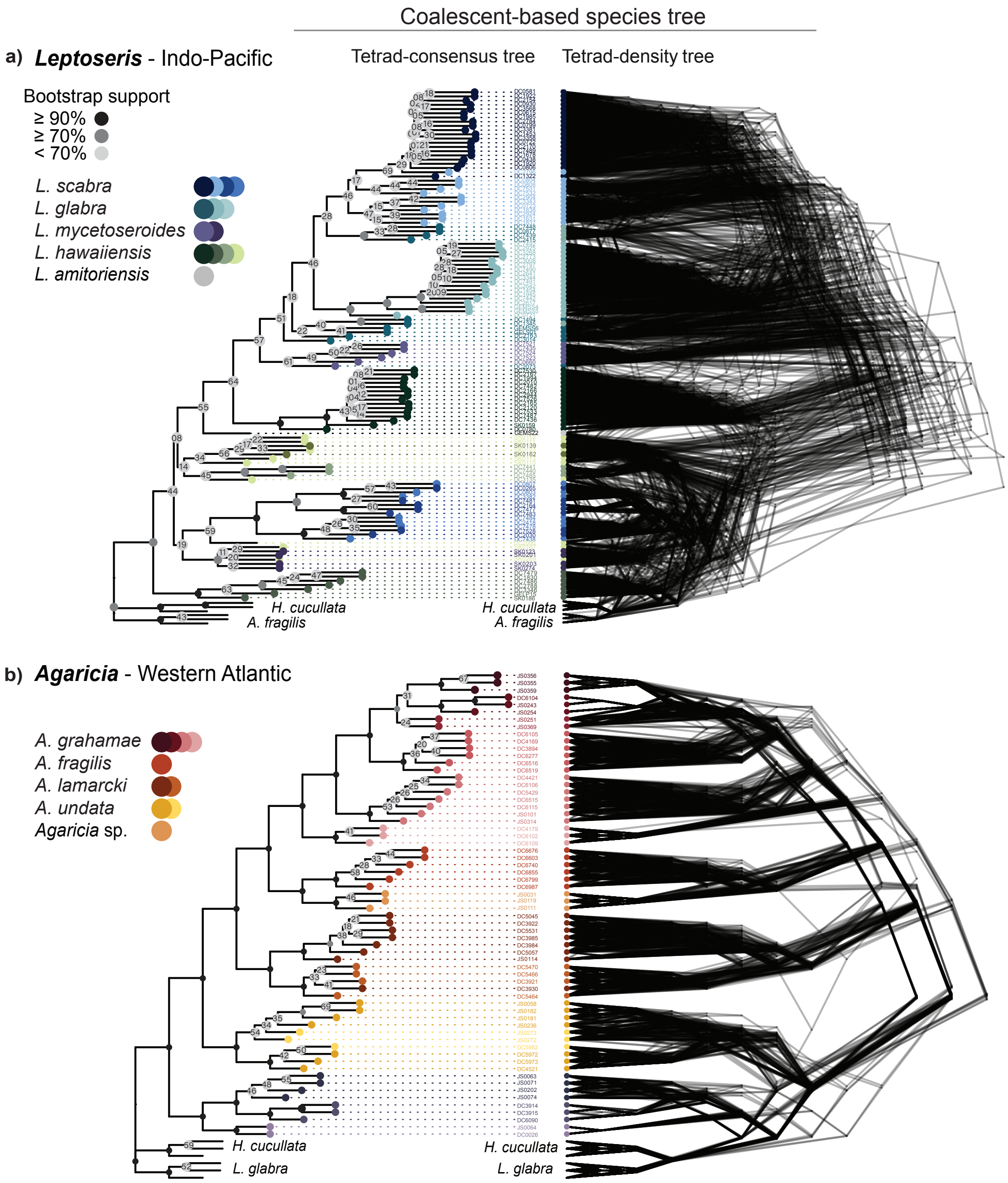

Supplementary figure S5. Evolutionary relationships of mesophotic *Leptoseris* and *Agaricia* species. (a) *Leptoseris*: Coalescent-based species tree: Tetrad; consensus tree of 100 bootstrap replicates (left), density tree visualizing 100 bootstrap trees (right). (b) *Agaricia*: Coalescent-based species tree: Tetrad; consensus tree of 100 bootstrap replicates (left), density tree visualizing 100 bootstrap trees (right).

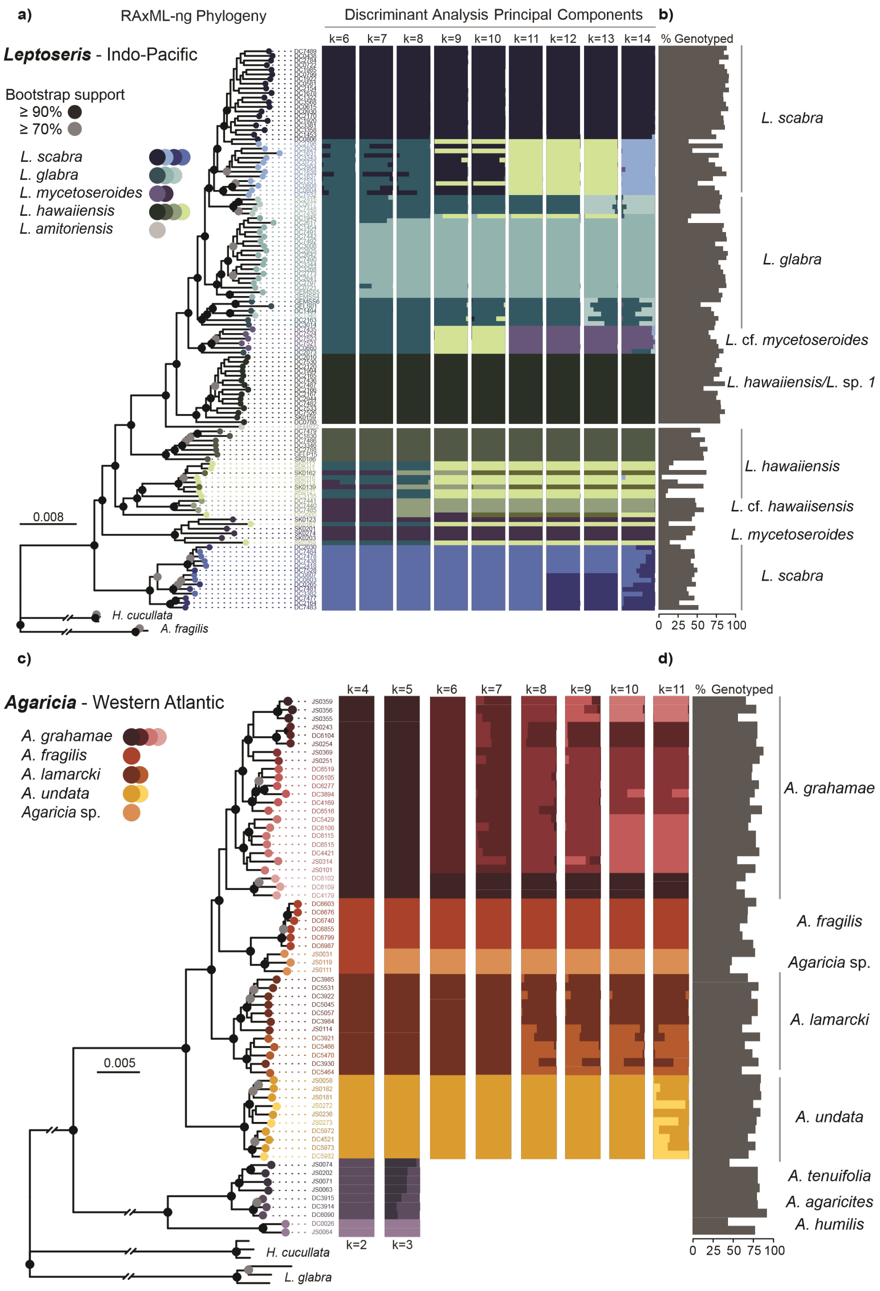

Supplementary figure S6. *De novo* clustering and ordination methods to assess genetic structure within the genus *Leptoseris* and *Agaricia*. (a) RAxML-NG tree of the genus *Leptoseris* based on concatenated nextRAD loci, next the posterior membership probabilities of the *de novo* discriminant analysis of principal components (DAPC) based on k=6 to k=14. (b) Barplot representing the percentage of genotyping across *Leptoseris* individuals. (c) RAxML-NG tree of the genus *Agaricia* based on concatenated nextRAD loci, next the posterior membership probabilities of the *de novo* DAPC based on k=6 to k=11, and k=2 to k=3 for the "*Undaria*" group (B) Barplot representing the percentage of genotyping across *Agaricia* individuals.

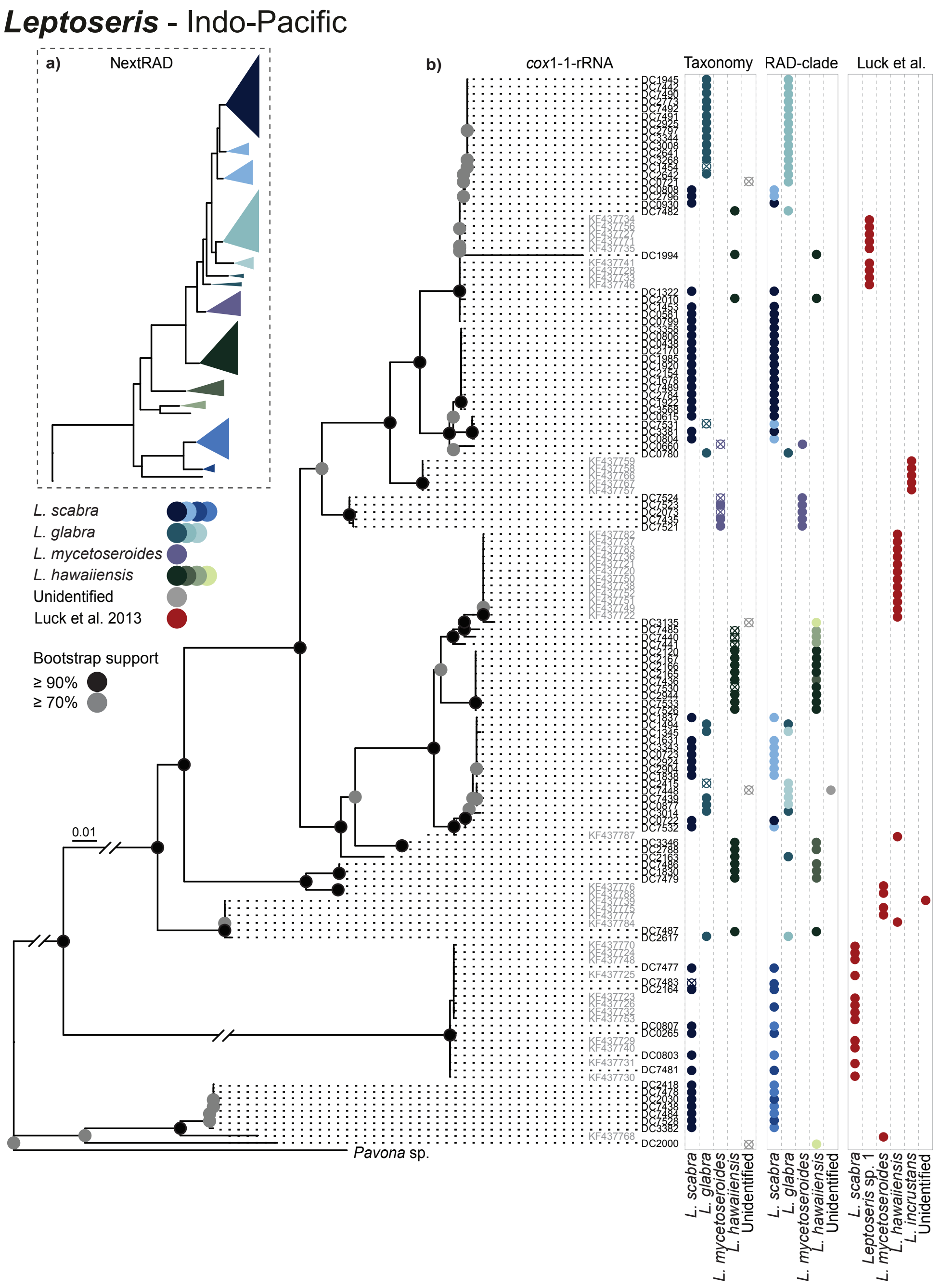

Supplementary figure S7. Comparison of maximum-likelihood based phylogenies of Australian and Hawaiian *Leptoseris* specimens. (a) RAxML-NG tree with collapsed nodes of the genus *Leptoseris* based on concatenated nextRAD loci, (b) Mitochondrial *cox*1-1-rRNA marker of a subset (n = 102) of the Australian samples, and Hawaiian samples from Luck *et al.* [17] (grey labels, n = 46), using an individual of *Pavona sp.* as outgroup; next, the taxonomic identification and the nextRAD clade assignment based on the DAPC results.

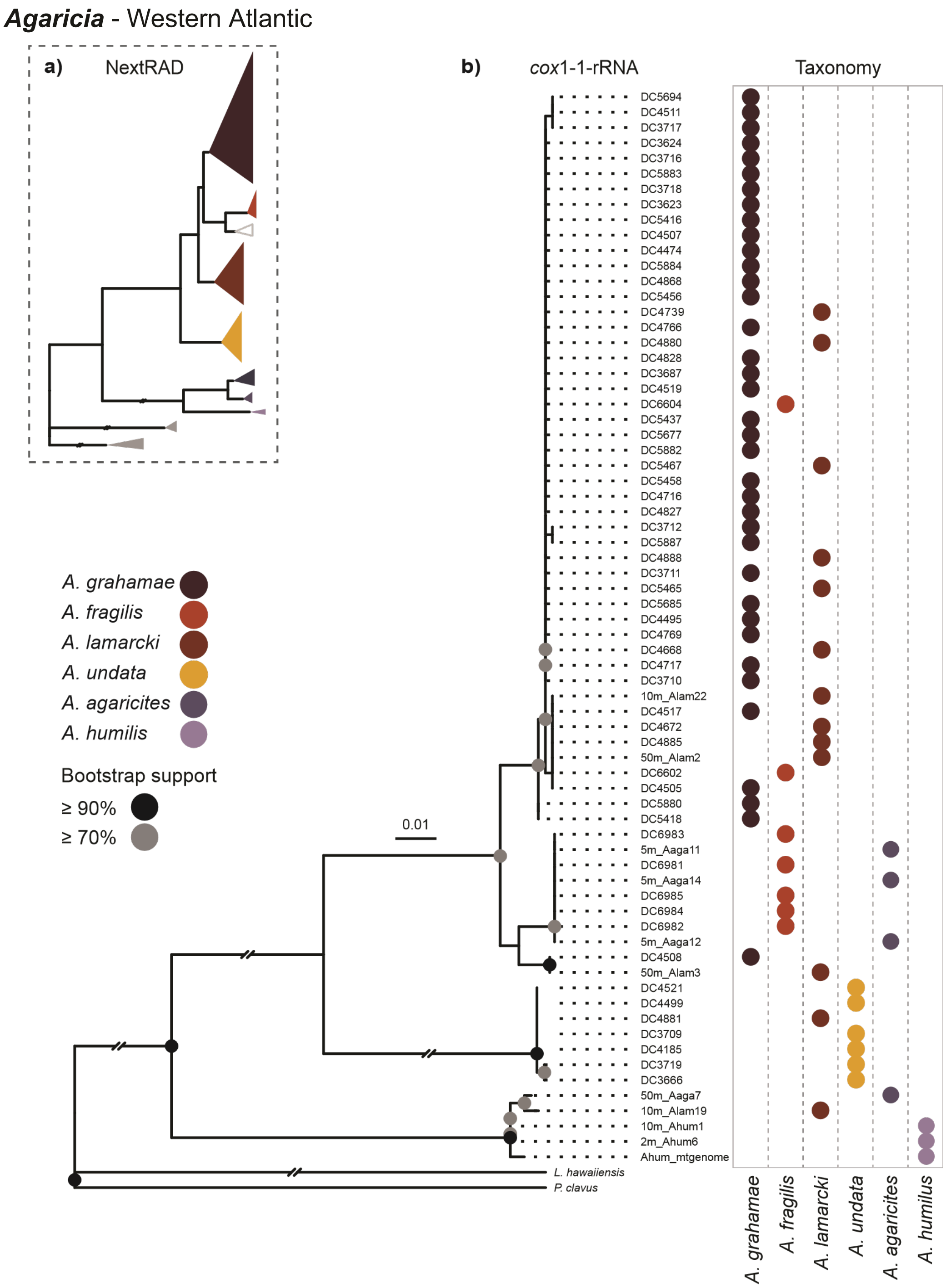

Supplementary figure S8. Comparison of maximum-likelihood based phylogenies of *Agaricia* specimens. (a) RAxML-NG tree with collapsed nodes of the genus *Agaricia* based on concatenated nextRAD loci from this study, (b) Mitochondrial *cox*1-1-rRNA marker of *Agaricia* individuals (n = 9) in addition to published *cox*1-1-rRNA sequence data of *Agaricia* individuals (n = 61) from Bongaerts *et al.* (2015) and Medina *et al.* (2006) with *Pavona clavus* (n = 1) and *L. hawaiiensis* (n = 1) as outgroup; next, the taxonomic identification.

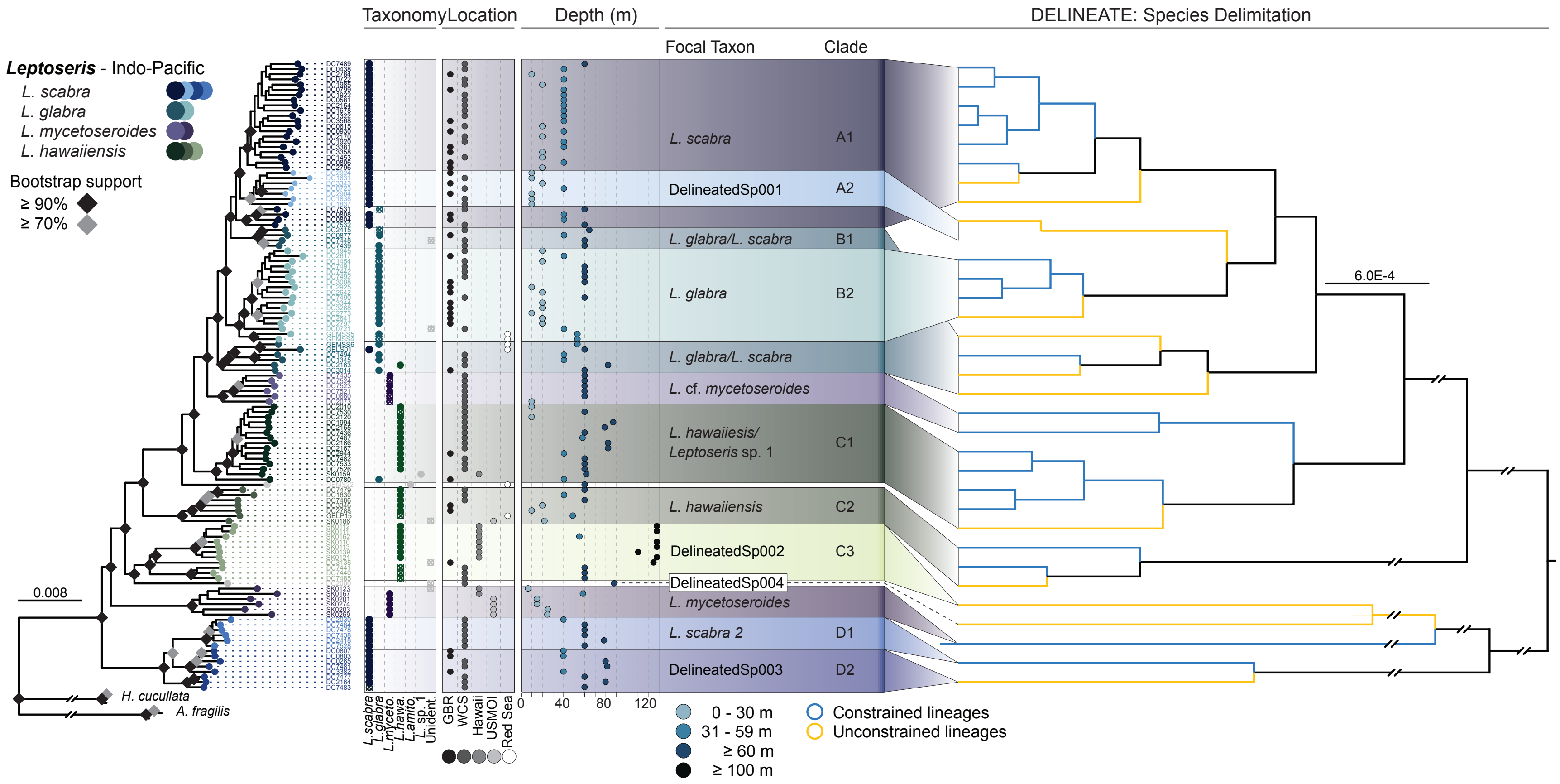

Figure S9. Species delimitation across the *Leptoseris* genus. Phylogenetic tree (RAxML-ng) of the genus *Leptoseris* based on 15,250 concatenated nextRAD loci (on the left). Background colours represent the different identified clades (based on the DELINEATE results) using blue, turquoise, purple, and green colours to represent the taxonomic species and shades for the different subclades. The tree on the right corresponds to a multi-population coalescent, ultrametric phylogeny where the blue branches indicate the constrained lineages assignment (i.e., constrained or "known" species), and the yellow branches represent the lineages with uncertain species identities (i.e., unconstrained).

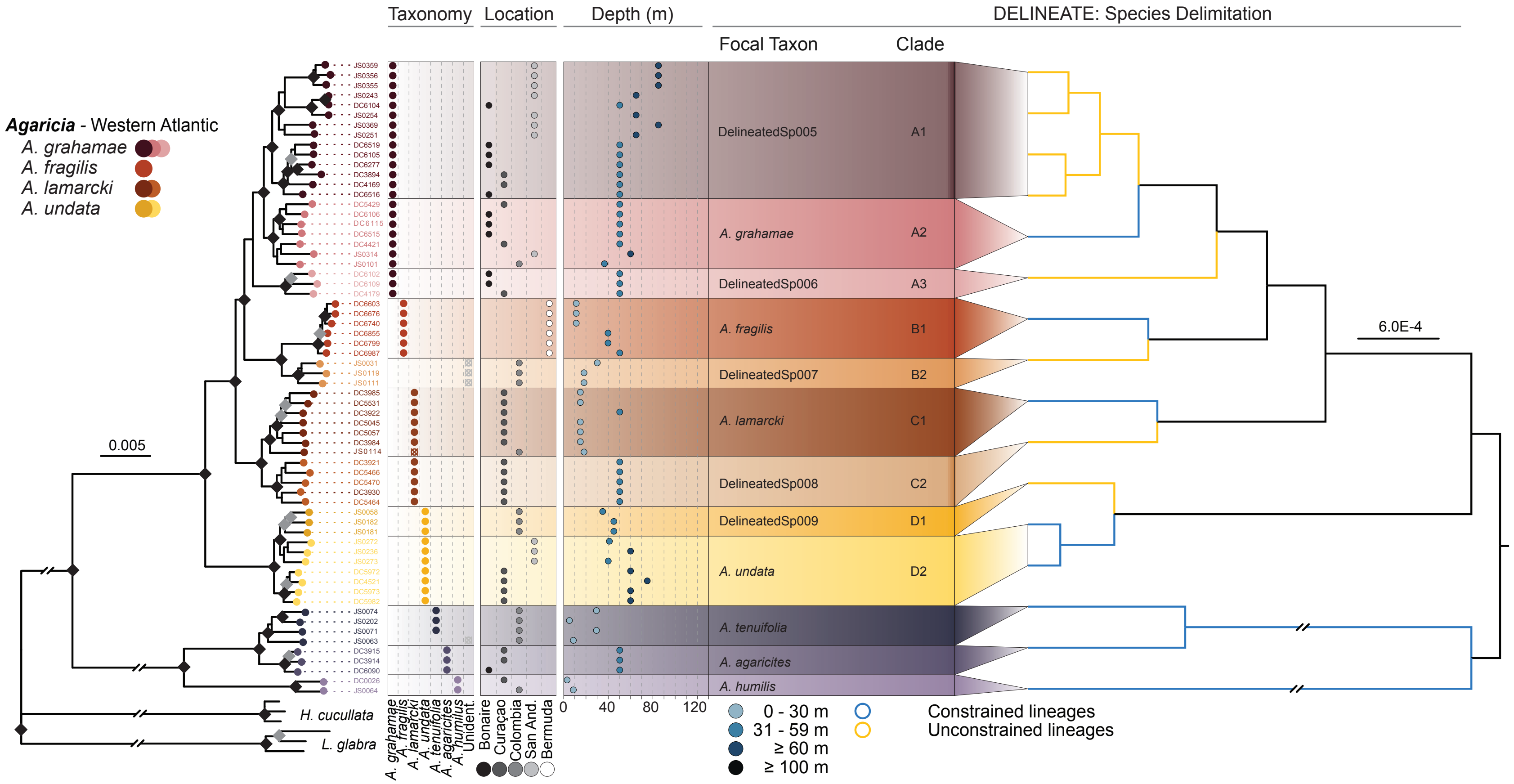

Figure S10. Species delimitation across the *Agaricia* genus. Phylogenetic tree (RAxML-ng) of the genus *Agaricia* based on 19,902 concatenated nextRAD loci (on the left). Background colours represent the different identified clades (based on the DELINEATE results) using dark red, orange, brown, and yellow colours to represent the taxonomic species and shades for the different subclades. The tree on the right corresponds to a multi-population coalescent, ultrametric phylogeny where the blue branches indicate the constrained lineages assignment (i.e., constrained or "known" species), and the yellow branches represent the lineages with uncertain species identities (i.e., unconstrained).
